# Guardian of Genetic Messenger-RNA-Binding Proteins

**DOI:** 10.3390/biom6010004

**Published:** 2016-01-06

**Authors:** Antje Anji, Meena Kumari

**Affiliations:** Department of Anatomy and Physiology, College of Veterinary Medicine, Kansas State University, Manhattan, KS 66506, USA; mkumari@vet.ksu.edu

**Keywords:** RNA-binding proteins, mRNA stability, mRNA splicing, gene expression, alcohol, neurological diseases, cancer

## Abstract

RNA in cells is always associated with RNA-binding proteins that regulate all aspects of RNA metabolism including RNA splicing, export from the nucleus, RNA localization, mRNA turn-over as well as translation. Given their diverse functions, cells express a variety of RNA-binding proteins, which play important roles in the pathologies of a number of diseases. In this review we focus on the effect of alcohol on different RNA-binding proteins and their possible contribution to alcohol-related disorders, and discuss the role of these proteins in the development of neurological diseases and cancer. We further discuss the conventional methods and newer techniques that are employed to identify RNA-binding proteins.

## 1. Introduction

In this review we provide a succinct overview of the role of RNA-binding proteins (RBPs) in regulating gene expression. Our summary is by no means exhaustive but is meant to give a flavor of the repertoire of RBPs recognized as important players in different disease processes. We have divided the review into subheadings under which we discuss the effect of alcohol on RNA-binding proteins, RNA-binding proteins in neurological diseases and cancer, and finally the methods employed to identify RBPs and/or ligands of a RBP.

**Figure 1:**
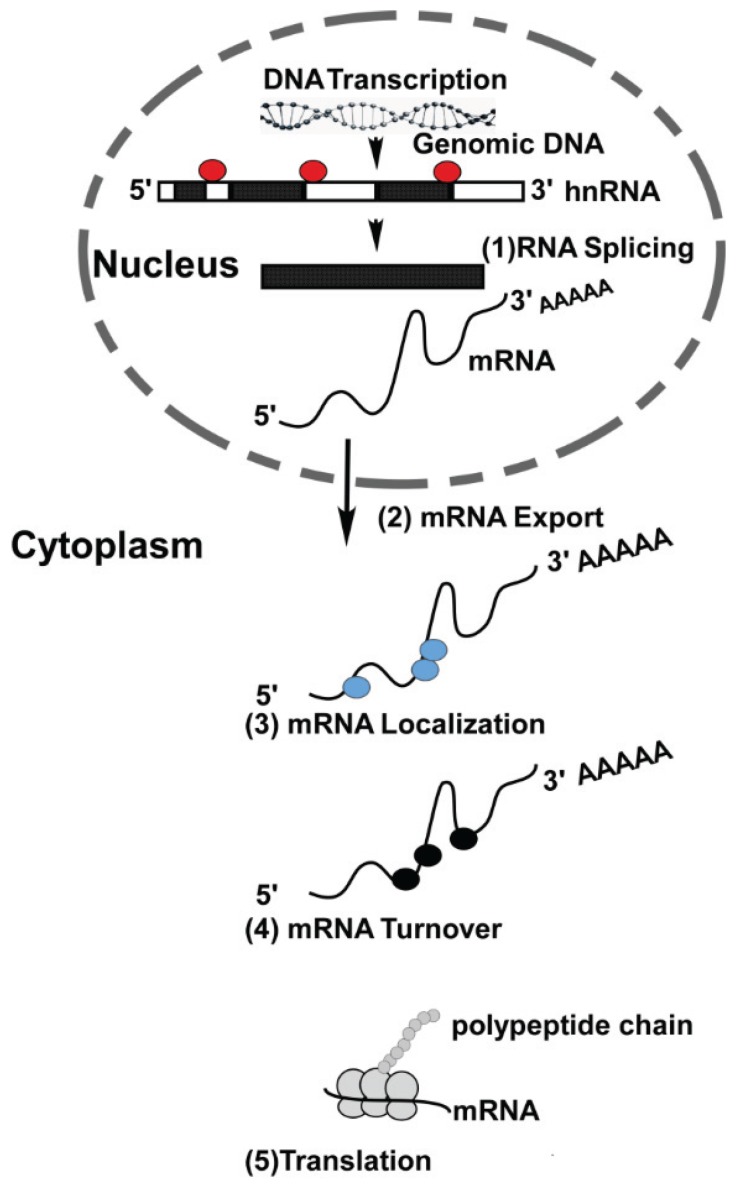
RNA-binding proteins and their role in RNA metabolism: Genomic DNA is transcribed in the nucleus resulting in generation of hnRNA. RNA-binding proteins (RBP) are involved in (**1**) splicing and alternative splicing of hnRNA, resulting in the formation of mRNAs. Messenger RNAs are then transported from nucleus into the cytoplasm (**2**). In the cytoplasm, RBPs aid in the localization of mRNAs to their final destination site (**3**). Once in the cytoplasm, each mRNA has a specific turn-over rate (**4**) that can be modulated by association with selective RBPs. The turn-over rate of mRNAs in cells can be altered in response to intrinsic and extrinsic stimuli. Messenger RNAs serve as a template for translation and RBPs play an important role in translation (**5**).

Gene expression is comprised of a series of well-coordinated events both at the transcriptional and post-transcriptional level. While we have a fairly good understanding of transcriptional events, post-transcriptional processes are comparatively far less understood. Transcription takes place in the nucleus where genes are transcribed from DNA, the blueprint of the cell. The transcribed RNA now represents information required to direct cellular function to maintain cell homeostasis. Before this information can be relayed to the cytoplasm, the nascent RNA undergoes extensive post-transcriptional processing before arriving at its final destination in cells. For this process the RNA is associated with nuclear proteins that aid in splicing and nuclear export [1]. As mRNA molecules emerge from the nucleus, bound nuclear proteins are often replaced with a new set of RNA-binding proteins (RBPs). In fact RBPs are involved in every step of the RNA lifecycle, e.g., transport of mRNA to the site of translation, storage and mRNA degradation (Figure 1) [1].

From the diversity of their function it is apparent that these proteins have an important role to play in most if not all post-transcriptional events. With the advent of high throughput screening techniques and quantitative proteomics, several hundred potential RBPs have been identified [2,3]. Given this large number of proteins, every effort is being made to expand our knowledge on RNA-protein interactions. RNA-binding proteins can be classified according to their pattern of expression and the type of target RNA. The pattern of RNA-binding protein expression can differentially regulate gene expression and this in turn can determine the fate of cells during development [4]. Expressions of target can be dampened or enhanced by RBPs as seen in yeast [5]. There are those proteins that are ubiquitously expressed and are associated with several different RNAs, while others are less promiscuous and associate with only a limited number of RNAs in specific cells. Messenger RNAs bind RBPs through one or more RNA-binding domains (RBDs). These domains are very versatile in that they can recognize either single or double stranded RNA, specific sequences in the primary structure of the RNA, or even RNA structures at the three dimensional level [6]. Compared to the large number of RBPs that have been identified or are predicted to serve as RBPs, relatively few RBDs have been identified so far although their number is constantly increasing [7]. Some of the RBDs are found only in a single species, whereas others are evolutionary conserved (for a review on RBDs see [7]). Most RBPs contain multiple RBDs. This arrangement has several distinct advantages. By utilizing several RBDs, the protein can recognize much longer stretches of nucleic acids. This scenario would not be applicable to a RBP harboring a single RBD [8]. Another advantage of having multiple RBDs on a single protein is the affinity of RNA-protein interaction. Proteins bind to target RNAs with high affinity. The presence of multiple RBDs allows for greater binding specificity. This is an important consideration given the observation that many more RBPs have been identified than RBDs. Multiple RBDs also make room for protein-protein interactions among RBPs, thereby increasing the stability of multiple RBPs-RNA complex and RNA-binding specificity of RBPs [8].

In an attempt to organize the expanding data on RBPs, Gerstberger *et al.* have compiled a census of human RBP [9]. They identified RBPs if proteins contained domains known to directly interact with RNA. Using this criterion, 1542 RBPs were manually curated and analyzed for their interaction with different classes of RNA, their evolutionary conservatism, abundance and tissue-specific expression. In another study 1344 genes encoding for RBPs in the human genome were catalogued [10]. Because many RBP lack bona fide RNA-binding motifs, Ray *et al.* set out to systematically analyze RNA motifs recognized by a diverse group of RBPs. The results of their study provide an important resource for post-transcriptional regulatory processes [11].

MicroRNAs (miRNAs) have emerged recently in the landscape of post-transcriptional regulation. They are considered both friends and foes due to their mode of action. A brief introduction to microRNAs and their mode of action is provided here. MicroRNAs are short (~22 nucleotides long) non-coding RNAs that are expressed in a tissue specific manner and bind specifically to target mRNAs. If RBPs are considered guardians of genetic messengers, then microRNAs may be referred to as “micro” guards of mRNAs. MicroRNAs control gene expression by the down regulating and fine tuning of transcripts by translational repression and/or degradation of respective mRNAs [12,13,14]. Regardless of the mechanism of miRNA operation, miRNAs hybridize to specific short sequences in the 3'-UTR of mRNAs [15]. A public repository (http://www.mirbase.org) has been created for all published miRNA sequences [16]. Since miRNA and mRNA interaction is sequence specific it is hard to predict mRNA target(s) of a miRNA. To overcome this shortcoming, several groups of researchers have developed computational methods to identify miRNA target sites in transcripts [17]. As both RBPs and miRNA associate with sequences in 3'-UTR of mRNAs, the question arises whether these regulators bind to the same sequence in the 3'-UTRs. Available data shows that some miRNAs compete for the RBP binding site, while the majority of experimental data suggests that miRNA-binding sites surround RBP-binding sites in the 3'-UTRs [15,18]. Databases such as starBase (http://starbase.sysu.edu.cn/) have been developed to identify mRNA-miRNA (+ other non-coding RNAs) and mRNA-protein-RNA interaction networks [19]. Simultaneous binding of miRNA and RBPs to their respective sequences in the mRNA can be co-operative where both molecules work in harmony or antagonistically to oppose their respective functions [14].

## 2. RNA-Binding Proteins and Alcohol 

Alcohol abuse poses a large disease with a high economic and social burden nationwide. In 2006 alcohol-related costs in the United States incurred the country $223.5 billion. Globally, 3.3 million deaths have been attributed to alcohol use [20]. Alcohol consumption affects many organ systems in the body and can change the pattern of gene expression both in culture and in animal models. Such alterations to gene expression are due to the consequences of alcohol’s ability to induce changes to normal cellular signaling, leading to changes in gene transcription. For instance, transcription factors and nuclear factors NF-κB and AP-1 are regulated both positively and negatively in pancreatic acinar cells following alcohol exposure. Such regulation is believed to contribute to ethanol toxicity in the pancreas [21]. Alcohol also modifies epigenetic landscape in cells, resulting in the modulation of gene expression [22]. In the central nervous system (CNS), alcohol has a pervasive effect and affects a number of neurotransmitter and neuromodulator systems. Some of the molecular changes associated with long-term effects of alcohol exposure are linked to alterations in signaling of the gene transcription factor, cAMP response element-binding protein (CREB) [23]. The consensus cAMP response element to which CREB binds has been identified in the promoter region of many genes [24], thus assigning this transcription factor an important role in the development of alcohol addiction [25]. While changes in epigenome, gene transcription, phosphorylation and signaling cascades in response to alcohol exposure have remained a focus of research, studies addressing the effects of alcohol on RBPs have been comparatively scant. One possible explanation for this difference may be the non-global cumbersome approaches of the “bygone era” to examine post-transcriptional events in the regulation of gene expression. We hope that the technological advancements such as CLIP and RNA-seq will stimulate more investigations in this exciting field of gene transcription. Nevertheless, with the help of enduring “gold standard” techniques of the “bygone era”, a number of studies have paved the path in this field. For example, it has been demonstrated that alcohol exposure does affect expression of RBPs, leading to disruption of molecular pathways in different cells/tissues.

**Figure 2:**
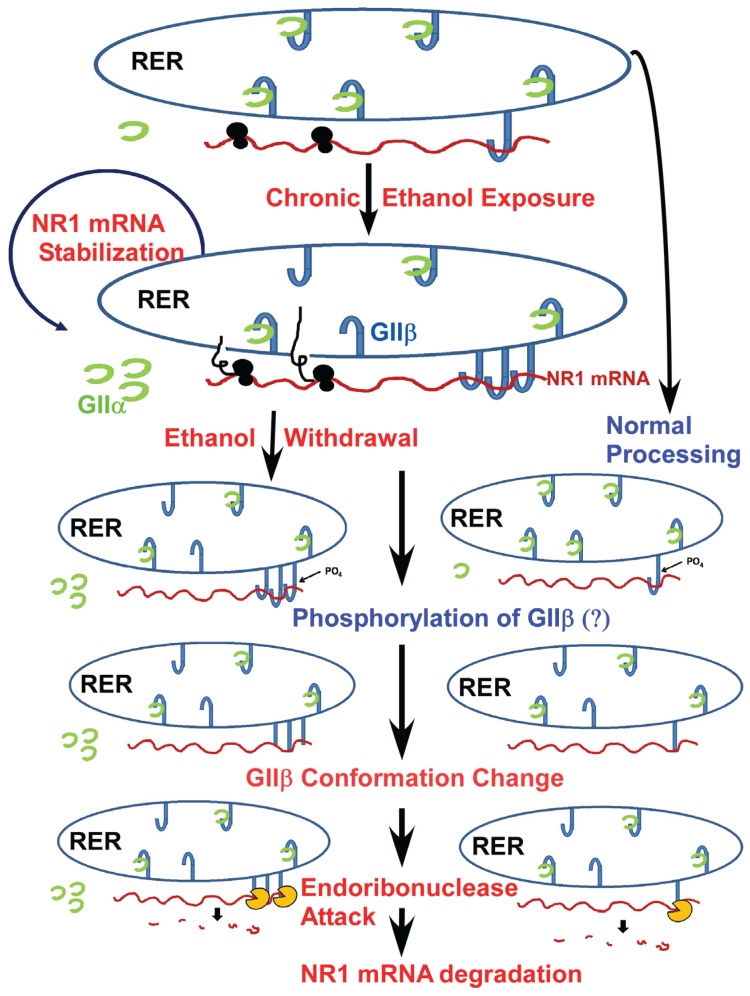
A proposed mechanism of NR1 mRNA regulation in normal and chronic ethanol exposed fetal cortical neurons: The NR1 mRNA is localized in the rough endoplasmic reticulum (RER) irrespective of ethanol treatment [36]. The normal half-life of NR1 mRNA is ~15 h in cultured fetal cortical neurons (FCNs). Following chronic ethanol exposure, the half-life of NR1 mRNA increases to more than 24 h in FCNs [35]. One of the potential mechanisms that can explain increase in NR1 mRNA half-life is increased binding of GIIβ to NR1 mRNA as demonstrated previously by us [38,39]. Decline in ethanol concentration, e.g., ethanol withdrawal in chronic ethanol exposed FCNs, triggers post-translational modification, e.g., phosphorylation of GIIβ—a process that occurs in normal FCNs as well. Phosphorylation of GIIβ alters the protein conformation such that GIIβ is no longer bound to NR1 mRNA. Loss of interaction with GIIβ allows NR1 mRNA to become temporarily “naked”. Endoribonclease(s) present in the vicinity are now able to attack the NR1 mRNA, eventually leading to NR1 mRNA degradation.

The glutamatergic neurotransmitter system is one major neurotransmitter system that is affected by alcohol exposure [26]. *N*-methyl-D-aspartate receptors (NMDA) belong to the family of glutamatergic receptors and are a well-established target of alcohol. They have been implicated in the development of alcohol dependence, tolerance, and withdrawal syndrome [27]. NMDA receptors assemble to form multimeric complexes consisting of different subunits. Molecular cloning revealed that NMDA receptors are comprised of seven different subunits, which fall into three distinct subfamilies: the GluN1 subunit, the four distinct GluN2 subunits (GluN2A, GluN2B, GluN2C, GluN2D) and two GluN3 subunits (GluN3A and GluN3B) [28]. The GluN1 subunit is considered the obligatory subunit, as functional receptors cannot be formed without it [29]. This subunit is the product of a single gene and has 8 full-length splice variants [30]. Chronic alcohol exposure induces a number of adaptive processes in the nervous system, including an upregulation of NMDA receptor number and function both *in vitro* and *in vivo* [31,32,33] with a concomitant increase in GluN1 and GluN2B receptor subunit expressions [33,34]. Our laboratory made the seminal observation on the GluN1 mRNA stability in cultured mouse fetal cortical neurons that are chronically exposed to alcohol. We found that the half-life of GluN1 mRNA increases to more than 24 h [35]. However, when the new protein synthesis is inhibited by culturing mouse fetal cortical neurons in the presence of the protein synthesis inhibitor, cycloheximide, the half-life of the GluN1 mRNA returns to control values [36]. This observation strongly suggested that new protein synthesis is critical to observe the increase in half-life of GluN1 mRNA. As it is well established that RNA-binding proteins contribute to the regulation of mRNA half-life [1], our laboratory undertook a systematic study to identify GluN1-mRNA-binding proteins that may regulate the half-life of GluN1 mRNA. Our investigations revealed a 156 nucleotides long *cis*-acting region in the 3'-UTR of GluN1 mRNA, and three RBPs bind to this region. Two of these RBPs are identified as annexin A2, a known RBP [37], and the second as the β subunit of α glucosidase II (GIIβ). In agreement with our observation that new protein synthesis is obligatory for the increase in GluN1 mRNA half-life to occur, we demonstrated that chronic alcohol exposure increases the expression of GIIβ both in cultured fetal cortical neurons and in the cerebral cortex of adult mice chronically exposed to alcohol [38,39]. The GIIβ protein is a novel RBP, as it was not known to bind RNA previously. GIIβ is the non-catalytic subunit of the α-glucosidase II (GII) [40]. GII enzyme is an ER resident protein, and its subunits alpha and beta are covalently linked [40,41]. The catalytic activity of the enzyme is conferred by the alpha subunit. The alpha subunit is a soluble protein and lacks an ER retention signal. It is retained in the ER by its association with the GIIβ subunit (Figure 2). Alpha glucosidase GII is an important enzyme in chaperone-assisted folding of nascent glycoproteins in the rough endoplasmic reticulum [40]. The notion that enzymes can serve as RBPs is not unlikely, as several metabolically active enzymes are known to interact with specific mRNAs [42]. For example, the enzyme glyceraldehyde-3-phosphate dehydrogenase (GAPDH) is originally identified as an enzyme important in glycolysis. However, further studies revealed that this enzyme also plays a role in other cellular processes [43]. GAPDH interacts with acts colony-stimulating factor-1 mRNA and regulates its half-life in ovarian cancer cells [44]. A microarray screen for RBPs in yeast revealed that a large proportion of previously known enzymes also act as RBPs [45]. Multifunctional enzymes are beneficial to an organism in that they can facilitate communication and/or cooperation between different cellular functions and pathways within the complex cellular network in cells.

The second GluN1 mRNA-binding protein, annexin A2 is a known RBP. Annexin A2 belongs to a large family of proteins that bind anionic phospholipids in a calcium dependent manner and are important in membrane organization and cytoskeletal interactions [46]. First, demonstration that annexin A2 is a RBD was documented in 2004. It was shown to bind specifically to c-myc RNA [47]. Later, it was found to interact with its own mRNA [37]. Binding of annexin A2 to *c-myc* and its own mRNA occurred via a putative RNA-binding domain consisting of five nucleotides 5'-AA(C/G)(A/U) [37]. This RNA-binding motif is also present in the 3'-UTR of GluN1 mRNA [39]. It appears that annexin A2 interacts with specific mRNAs translated on cytoskeletal-bound polysomes [24]. Indeed the GluN1 mRNA is always associated with polysomes. However, the exact role of annexin A2 as RBP has not been elucidated. It is likely that it is involved in mRNA transport and localization.

Binge drinking refers to consumption of 4–5 drinks* (as ≥4 drinks for a woman and ≥5 drinks for a man on a single occasion) within 2 h as per NIH-NIAAA guidelines, and this type of drinking brings blood alcohol levels to 0.08 g/dL. According to NIH-NIAAA, a standard drink in the US is considered as 12 ounces of regular beer (about 5% alcohol) or 5 ounces of wine (about 12% alcohol) or 1.5 ounces of 80-proof distilled spirits (about 40% alcohol) [20]. Binge drinking has become a popular form of alcohol abuse in the younger population and more recently in the older generation [48]. In younger individuals, the brain is particularly sensitive to the repeated withdrawals of alcohol resulting from alternating periods of intoxication and withdrawal. Binge drinking has serious consequences, such as impairment of the cognitive function [49]. One of the mechanisms proposed for a decline in cognitive function in binge drinkers is the inflammation of central nervous system [50]. Resident macrophages in the brain are called microglia, and they constitute a large proportion of cells in the brain. Microglia perform a number of functions in the context of inflammation and brain injury [51]. Activation of microglia is considered to be neuroprotective. However, over-activation of microglia results in the release of neuroinflammatory molecules that subsequently lead to neuronal pathology [52]. Recently, a protein with a molecular mass of 18 kDa was identified as a candidate protein that could facilitate alcohol-induced neuroinflammation [39]. The 18 kDa protein is a cold shock protein with an N-terminal RNA-binding domain. This protein is named as cold inducible RNA-binding protein (CIRP) because its expression at the mRNA and protein level can be induced within 12 h after lowering the temperature from 37 °C to 32 °C [53]. CIRP is involved in a number of cellular processes including transcription, translation, DNA recombination, and cell proliferation [53]. In most tissues and cell lines, CIRP is constitutively expressed albeit in low amounts [54]. However, a significant increase in the expression of CIRP mRNA and protein is noted in the mouse brain after binge alcohol intoxication. Similar effects of alcohol are observed on CIRP expressions in the cultured murine microglial cell line BV2 [55]. In CIRP knock-out (CIRP^−/−^) mice, levels of inflammatory molecules such as TNFα and IL-1β are markedly attenuated as compared to wild type mice following alcohol consumption. Thus, both *in vivo* and *in vitro* studies assign a new function to CIRP of being a proinflammatory molecule that mediates inflammation in the brain following alcohol consumption [55]. Recent studies show that CIRP modulates a circadian gene called the Circadian Locomotor Output Cycles Kaput (CLOCK) expression post-transcriptionally in mammalian tissues [56]. A normal expression of CLOCK genes is necessary for maintenance of mammalian circadian function. An increased expression of CIRP following binge drinking can disrupt circadian function, and this may lead to stress and sleep rhythm disturbances [57].

Alcohol does not only affect the mature brain but also has profound effects on the developing brain. Maternal alcohol exposure during pregnancy results in a number of abnormalities collectively termed fetal alcohol syndrome (FAS). At the cellular level, FAS is characterized by neurodegeneration mainly as a result of apoptosis [58]. In the developing brain, the balance between cell differentiation and apoptosis is tightly regulated so that the large number of neuronal cells can make proper connections with other cells and that synaptogenesis can occur in an orderly fashion. The RNA-binding protein, Napor, which was first identified in the fetal heart [59], is actually involved in apoptosis in the developing brain as it is highlighted in screens of genes induced during apoptosis in neuroblastoma cells [60]. Napor is linked to nuclear and cytoplasmic RNA processing events such as alternative splicing and mRNA stability [61,62]. Using a differential display polymerase chain reaction and RNase protection assays, Naha *et al.* observed a reduction in Napor expression in forebrain, midbrain and hindbrain of fetal rats exposed to alcohol during development [63]. Considering Napor’s role in programmed cell death, RNA splicing and stability, any alterations in its expression could conceivably disrupt the formation of correct neuronal connections and cellular homeostasis. Neuronal homeostasis is critical for normal neuronal development. Therefore, a decrease in Napor may contribute to apoptosis mediated neurodegeneration—the hallmark of FAS.

Alcohol affects almost all tissues in our body. The liver is another organ that has been examined for changes in expression of RBPs, as altered expression of RBPs disrupts molecular pathways in different cells and tissues. The RBP HuR is a member of the embryonic lethal abnormal vision (ELAV) family of RBPs originally identified in *Drosophila* as an essential protein for neural development [64]. HuR is ubiquitously expressed and plays a role in muscle differentiation and adipogenesis, and promotes apoptosis [65]. Tumor necrosis factor alpha (TNFα) is a key regulator of the inflammatory response and is produced mainly by activated macrophages and T-lymphocytes. It is thus not surprising that its expression is increased in chronic inflammatory diseases such as alcoholism [66]. TNFα expression is tightly regulated both at the transcriptional and at the post-transcriptional level [67]. HuR is an important post-transcriptional regulator of TNFα. HuR specifically binds to the AU-rich element (ARE) located in the 3′-UTR of TNFα mRNA in macrophage-like cell line, RAW264.7 [68]. The AU rich elements located in the 3′-UTRs of many mRNAs represent the most common mRNA instability element [69]. The binding of proteins to ARE protects mRNAs from degradation. Hence ARE-protein interaction(s) is a pre-requisite for mRNA stability and translation as in the case of TNFα [70]. An increased expression of TNFα is implicated in the pathogenesis of alcoholic liver disease [71]. The main reason for associating TNFα with alcoholic liver disease is that TNFα expression is significantly increased following chronic alcohol exposure in laboratory rodents. Increase in TNFα expression is caused by lipopolysaccharide (LPS) stimulation of Kupffer cells in liver. Upon LPS stimulation, the half-life of TNFα mRNA increases due to a binding of HuR to an ARE sequence in the 3'-UTR of TNFα mRNA [68]. It is also noted that the RNA-binding activity of HuR is increased after the chronic alcohol exposure of Kupffer cells [72].

Liver disease is a major and debilitating consequence of chronic alcohol consumption. Excessive alcohol exposure can lead to hepatosteatosis and to more severe consequences such as liver fibrosis and cirrhosis. How alcohol brings about this effect has been the focus of much investigation. Galβ1,4GlcNAc-α2,6-sialyltransferase (ST6Gal1) mediates the addition of α2,6-linked sialic acid to glycoproteins in the Golgi apparatus. It was observed that the mRNA levels of ST6Gal1 are reduced in post-mortem human livers of moderate and heavy alcohol drinkers. Subsequent studies involving laboratory rodents showed a significant decline in ST6Gal1 activity accompanied by reduction in ST6Gal1 mRNA levels following chronic alcohol exposure [73,74]. The importance of this finding lies in the fact that the reduction in ST6Gal1 enzyme activity causes faulty glycosylation of lipid transporters, apolipoproteins apoE and apoJ. Improper glycosylation of apolipoproteins apoE and apoJ prompts perturbations of intracellular lipid transport [75]. This was a compelling reason to further investigate molecular events causing reduction of ST6Gal1 enzyme activity. The use of techniques such as EMSA and UV crosslinking were crucial in identifying interaction between 3'-UTR of ST6Gal1 mRNA and a cytosolic protein with a molecular mass of 41 kDa. The 41 kDa protein was detected in both rat livers and post-mortem human livers of moderate and heavy alcohol drinkers [74]. The identity of the 41 kDa protein is not known at this time. However, it is believed that 41 kDa cytosolic protein plays a role in ST6Gal1 mRNA stability as its intracellular concentration decreases following alcohol exposure. Importantly, the relative intensity of the RNA-protein complex decreases with an increase in alcohol consumption, suggesting that the 41 kDa RBP associating with the 3'-UTR of ST6Gal1 mRNA is an alcohol responsive protein.

## 3. RNA-Binding Proteins in Neurological Diseases

RBPs are ubiquitous partners of RNA and control all aspects of RNA metabolism. Given their omnipresent nature, dysregulation of RNA processing in a number of pathologies ranging from neurologic diseases to muscular dystrophies and cancer places them on a higher pedestal. Thus, the pathologies have become a target of novel therapeutic drugs [76,77,78]. Among the disorders attributed to mutations in RBPs, neurologic diseases feature most prominently. One reason why RBPs play such a prominent role in CNS disorders is because RBPs play a central role in alternative splicing, and this post-transcriptional regulatory mechanism is most prevalent in the nervous system [79]. In addition, RBPs are essential to protect mRNAs from premature degradation and translation during their journey from their site of synthesis in the cell nucleus to cytoplasm, dendrites and axons for *de novo* protein synthesis [80]. These functions of RBPs create vast opportunities for these proteins to contribute to the pathogenesis of neurological diseases.

Paraneoplastic neurologic disorders (PND) are neurological manifestations resulting from autoantibodies against two well-known families of RBPs, the Nova and the Hu [81,82]. Family members of Nova and Hu are expressed ectopically by tumors in breast, lung and/or bladder [65,83,84]. Normally, these members of Nova and Hu are expressed only in the CNS, an immune privileged environment. However, when they are produced by tumors outside the CNS, they are recognized as foreign antigens, and an immune response is elicited against their resulting in generation of antibodies. These antibodies are classified as autoantibodies, as they are produced against an individual’s own protein. One such example is Paraneoplastic Opsoclonus Myoclonus Ataxia (POMA) [85]. In POMA patients, autoantibodies are generated as a result of breast, gynecologic, lung and bladder cancers. These antibodies target a neuron-specific Nova family of RBPs. Nova 1 is expressed primarily in the hindbrain and spinal cord, while Nova 2 is expressed mainly in the neocortex [86]. At least 34 target transcripts of Nova are identified using ultraviolet cross-linking and the immunoprecipitation (CLIP) technique [87]. The majority of Nova targets are involved in neuronal synaptic function, and only one third of the targets are involved in mediating inhibitory neurotransmission. Nova is important for alternative splicing of two inhibitory receptor mRNAs in the brain, glycine α2 and GABA_A_ [88]. Gephyrin mRNA that encodes a protein involved in clustering of both GABA and glycine receptors is also a Nova target [87]. As a consequence of the loss of Nova function, patients diagnosed with POMA display the dysregulation of inhibitory neurotransmission characterized by an excess of motor movements. Nova knock-out mice die 7–10 days after birth due to progressive motor dysfunction coupled with apoptotic cell death in NOVA-expressing brain regions [89]. The Nova knock-out mouse model was crucial in providing evidence for the importance of Nova in alternative splicing events. In a clinical setting, patients suffering from POMA have provided us with invaluable insight into the clinical consequences of Nova dysfunction.

The Hu family members (HuR, HuB, HuC, and HuD) are a second set of RBPs with diverse functions and mediate antigen-associated immune disorders. Two Hu family members, HuC and HuD are specifically expressed in the CNS. The Hu family of RBPs regulates half-lives of several mRNAs by binding to ARE regions in their 3'-UTRs. The binding of Hu family members to mRNAs, such as epidermal growth factor, *cFos* and granulocyte-macrophage stimulating factor, results in stabilization of the respective mRNA [90]. Because of having diverse targets, these RBPs are implicated in many aspects of neuronal functions. For instance, the inactivation of HuD results in impaired neural development with motor co-ordination defects in mice [91]. In contrast, overexpression of HuD leads to abnormal mRNA stabilization associated with cognitive deficits in mice [92]. Diverse neurological dysfunctions, such as sensory neuropathy, cerebellar ataxia and encephalitis, are exhibited by patients diagnosed with PND or the anti-Hu syndrome [82]. Molecular mechanisms underlying these diverse neurological dysfunctions were elucidated by a number of investigators in the field [65]. The majority of PNDs are associated with small cell lung cancer and gynecologic tumors. These cancer tissues ectopically express Hu family members such as HuB, HuC and/or HuD. An immune response is mounted in humans suffering from these cancers. Once the antibodies are generated, they enter the circulation and subsequently cross the blood-brain barrier. In the brain, these antibodies bind to HuC and HuD. Bioneutralization of HuC and HuD leads to dysregulation of several mRNAs. This in turn results in PND-associated neurological dysfunctions including encephalomyelitis and neuropathy [65].

In addition to modulating splicing events and regulating mRNA half-lives, RBPs play important roles in translational control during embryonic development, cell-cycle progression [93], learning, and memory formation [94]. Defects in translational control have profound effects on cellular functions resulting in a multitude of diseases ranging from cancer to autism. A classic example of a consequence of translational dysfunction is the Fragile X syndrome (FXS), a disease characterized by intellectual disability, autistic behaviors and childhood seizures [95]. Fragile X syndrome was the first genetic disorder to link RNA regulation to cognitive function. This syndrome is caused by the absence of the RBP, Fragile X mental retardation protein (FMRP). The protein is widely expressed in mammalian tissues but is particularly abundant in neurons where it can be found in the cell body, proximal dendrites and axons. FMRP is a component of messenger ribonucleoprotein complexes that are associated with polyribosomes [96]. Because of this association, FMRP is implicated in translational repression [97,98]. Translational repression in neurons is very important because of cell architecture. Neurons have a cell body with extensive dendritic arbors and axonal projections. Proteins are translated both in cell bodies and in dendritic spines [99,100,101,102]. Translation in dendritic spines is generally activity dependent [100,102,103]. Several mRNAs have been localized in dendritic spines [104]. Although the process of mRNA transport to dendritic spines is not completely understood, mRNAs are translationally repressed during their transport. Once they have reached their destination, mRNAs may remain in a repressed state until synaptic stimulation triggers protein translation. Translation outside of the cell body, locally at the synapse, is particularly important for the maintenance of synaptic plasticity and synaptic plasticity in response to stimuli that do not involve nuclear events. Recently, Darnell and colleagues demonstrated that translational repression by FMRP is achieved by stalling ribosomal translocation [97]. It is thus understandable that the loss of FMRP function results in defects of activity-dependent protein synthesis in dendritic spines, which leads to changes in synaptic plasticity [98].

## 4. RNA-Binding Proteins and Cancer

Cancer is a heterogeneous disease, and we have no clear understanding of the etiology of different tumors. Traditionally, it is believed that the development of cancer is associated with mutations, transcriptional dysfunction and signal pathways gone awry. However, as we gain a greater understanding of post-transcriptional regulation of gene expression, we realize that post-transcriptional events contribute to cell proliferation, differentiation, apoptosis and angiogenesis—all processes that modulate the development and progression of cancer [105]. RNA-binding proteins regulate all aspects of post-transcriptional gene expression of tumor expressers and oncogenes. It is therefore not surprising that RBPs play an important role in cancer. A recent study by Kechavarzi and Janga explored the expression pattern of RBPs in human cancers. They found at least 30 RBPs were upregulated in two-thirds of all the cancers examined [106]. Here, we focus on some RBPs that are important in cancer and participate in alternate splicing and protein translation. The protein, Src-associated in mitosis (Sam 68), is a member of the STAR (Signal Transduction and Activation) family of RBPs and is important in cellular differentiation and proliferation [76]. Sam 68 is overexpressed in breast and prostate cancer [107,108]. It is a predominantly nuclear protein involved in alternate splicing of cancer related mRNAs. For instance, CD 44 mRNA encodes a cell surface glycoprotein involved in cell-cell and cell-matrix adhesion in cancer cells [109]. Sam 68 regulates alternate splicing of CD 44 pre-mRNA generating more than 20 isoforms encoding for extracellular domains implicated in cancer development [109]. Cyclin D1 is a proto-oncogene involved in cell-cycle progression. Given its role in cell proliferation, it is tightly regulated. Overexpression of cyclin D1 is correlated with the development and progression of cancer [110]. Cyclin D1 has two isoforms D1a and D1b, generated by alternate splicing of exon 4. Both isoforms are frequently upregulated in cancers. However, out of the two, D1b isoform exhibits greater oncogenic properties. Interestingly, Sam 68 is also involved in splicing of cyclin D1 and promotes expression of cyclin D1b isoform [111].

The Hu family of RBPs is another example involved in the development of cancer. Of the Hu family of proteins, HuR is the one that is most frequently associated with the development of different cancers including breast, pancreatic, prostate, lung and ovarian cancer [112]. In a resting state, HuR is predominantly localized in the nucleus but translocates to the cytoplasm following post-translational modification. Once in the cytoplasm, HuR binds to mRNAs, harboring ARE sequences in their 3'-UTRs, resulting in stabilization of target mRNAs with an increase in half-lives of the respective mRNAs. Targets of HuR include Bcl2, CoXII, VEGF, certain kinases, and cyclins—all these target mRNAs are involved in cellular growth and proliferation [83]. An increase in half-life of these mRNAs can disrupt the tightly regulated homeostasis of cell proliferation, leading to the development of cancer.

Translation control is a critical constituent of cancer development and progression. During the multistep process of protein translation, the translation factor elF4E binds to the 7-methyl guanosine cap structure at the end of mRNAs, providing a critical interface for the recruitment of the 40S ribosomal subunit and other translation factors [113]. Expression of elF4E is controlled at multiple levels, and its increased activity has been implicated in different cancers [113]. It is interesting to note that increased expression of elF4E is not associated with a global increase in protein synthesis but appears to promote translation of mRNAs involved in oncogenesis. Such mRNAs have longer 5' untranslated regions with higher GC content, resulting in more complex hairpin structure formation—one of the mechanisms that provide control over the protein expression of cancer promoting mRNAs. By increasing the expression of elF4E, translation of these subsets of mRNAs may be facilitated, allowing for their selective activation, and thereby promoting tumor formation and progression. From the above highlights of RBPs role in cancer, it is clear that these proteins are powerful players in the development and progression of cancer. However, what is not clear at this time is whether alterations in expression of RBPs are a cause or a consequence of cancer.

## 5. Approaches to Detection of RBPs

Traditionally, *in vitro* methods have been utilized to detect RNA-protein interactions, and these methods remain in vogue. EMSA has been employed extensively in past years and has yielded invaluable information. In this assay, radiolabeled RNA was incubated with the source of RBP (purified protein or cell lysate or microsomes or membrane free cytosol), and the resulting complex was electrophoresed on a non-denaturing polyacrylamide gel and radiolabeled bands were visualized by exposure to X-ray film or PhosphorImager. Non-specific RNA-protein interaction was prevented by the incubation of RNA and protein source in the presence of tRNA and/or heparin. The principle behind this technique is the observation that naked RNA has certain mobility on gels, but its mobility is retarded upon binding to proteins. To confirm the specificity of the RNA protein interactions, an excess of unlabeled competitor RNA is added to the reaction mix, and this obliterates the formation of RNA-protein complex. To confirm the presence of a specific protein in the RNA protein complex, gel super shift assays can be performed. These assays were performed as in EMSA, except for the addition of specific antibody to the protein of interest. Formation of RNA-protein-antibody complex further slows down the migration of complex on a gel. Once the interaction between a specific mRNA and a RBP is established, immune-RT/PCR can be employed to confirm the RNA-protein interactions [38]. Briefly, the RBP of interest was immunoprecipitated following which RNA was extracted from the precipitate, and the RNA of interest was amplified using gene specific primers. Another widely used technique in RNA biology is the conventional UV-cross link analysis. This analytical technique takes advantage of the ability of short-wave ultraviolet light (254 nm) to activate nucleotide bases (e.g., uracil and to a lesser degree other nucleotides) to covalently bind to specific amino acids such as Cys, Lys, Trp and Tyr in proteins [114,115]. In this assay, the protein lysate and radiolabeled RNA were incubated to allow complex formation. The reaction mix was then UV-irradiated and treated with ribonucleases to digest unbound RNA. UV irradiation will covalently link RNA to proteins, but protein-protein interactions are not affected. The protein complex was subsequently resolved on SDS-PAGE. Gels were dried and the radiolabeled RNA-protein complexes were visualized as in EMSA. As opposed to EMSA where one can only document the ability of proteins to bind to a specific RNA, UV-cross link analysis yields information on the number of RBPs binding to a specific RNA or RNA fragment containing *cis*-acting region. The size of RBPs binding to RNA can be determined by northwestern analysis. In this technique, protein lysates are separated on SDS-PAGE and proteins are re-folded and then transferred to a support membrane such as nitrocellulose membrane. Proteins transferred to membrane were probed with radiolabeled RNA. Non-specifically bound RNA was washed off and the membrane was exposed to X-ray film to reveal the number of proteins highlighted by RNA. The molecular size of proteins was extrapolated from the standard molecular weight marker proteins separated along with protein samples on the gel.

The aforementioned *in vitro* techniques continue to be powerful tools in the RNA field. To detect RNA-protein interactions *in vivo*, Darnell *et al.* developed the cross-linking and immunoprecipitation (CLIP) method [116]. A distinct advantage of this method is the cross-linking of RNA-proteins in live cells and/or tissue preserving native associations in the absence of salt and ionic components that need to be optimized in *in vitro* assays. Another advantage of this technique is that one can identify target RNAs of one RBP. The CLIP procedure was comprised of a number of steps that we have briefly summarized here. For a detailed protocol, the reader is referred to the original publication [116]. In short, after the tissue or cells were UV-cross linked, lysate was prepared, which was treated with nucleases to trim the cross-linked RNA to 50–100 nucleotide size. The lysate was cleared of ribosomes and incubated with beads coupled to the antibody against the RBP of interest. RNA-protein complexes are partially purified by immunoprecipitation, and non-covalently associated RNA was removed by SDS-PAGE. The purified RNA protein complexes were treated with proteinase K to remove proteins but leave RNA, which was abundant enough to perform RNA linker ligation, RT/PCR amplification and sequencing. The resultant short (70–100 nucleotides) RNA CLIP “tags”, which represent native RBP binding sites, were able to be database-matched to identify the targets of an RBP. This technique allowed Darnell *et al.* to identify 34 targets of NOVA in the brain [116].

With the advent of high-throughput sequencing, it was then possible to map RNA-binding sites even more comprehensively. Using such a platform, millions of unique RNA tags were able to be identified and mapped back to the genome yielding transcriptome-wide RNA-protein binding maps. To further refine the CLIP protocol, Darnell *et al.* replaced the conventional Sanger sequencing method employed in CLIP with high-throughput sequencing (HITS-CLIP) [117]. This method allowed investigators to generate a transcriptome-wide understanding of NOVA-RNA regulation.

## 6. Computational Methods

RBPs interact with mRNA via a number of RNA recognition motifs or RNA-binding domains [7]. A large number of proteins have been predicted to serve as RBPs using these sets of RNA recognition motifs (http://rbpdb.ccbr.utoronto.ca/) [118]. In *Caenorhabditis elegans*, as many as 887 genes may encode for RBPs [119] and, in *Saccharomyces cerevisiae*, approximately 600 transcripts encode for putative RBPs [120]. As our knowledge on RBPs expands, it is becoming apparent that many RBPs do not harbor conventional or previously identified RNA recognition motifs. For instance, the novel GluN1 mRNA-binding protein, GIIβ does not contain a known RNA recognition motif, and conventional methods were employed in its identification as a RBP [38]. Computational methods can serve as powerful complementary approaches to identify RBPs. These methods for post-transcriptional analyses have recently gained importance as declining costs of next-generation sequencing have made global approaches more affordable. Some of the high-throughput assays currently employed by RNA biologists to study post-transcriptional regulation are CLIP, HITS-CLIP, PAR-CLIP and iCLIP [17]. The utility and advantages, as well as the disadvantages, of these approaches have been described [121]. In general, these techniques involve *in vitro* or *in vivo* cross-linking of RNA and proteins, digestion of unbound RNA, immunoprecipitation using an antibody to a RBP of interest, reverse transcription, PCR amplification, and high-throughput sequencing. High-throughput sequencing data is typically uploaded onto public databases such as the Gene Expression Omnibus (GEO from NCBI) and ArrayExpress (from EBI), and three recently developed databases for CLIP-seq: CLIPZ, doRiNA, and starbase. As a next step, using computational methods, such as Bowtie, RMAP, Novoalign, and TopHat, the “short reads” (sequencing data sets) are aligned to a reference sequence (transcriptome and genome) while eliminating technical sequencing errors and true genetic variations. Once the “short reads” are aligned to the reference sequence, several restrictions are applied to filter the noise in processing the data further to detect a cluster of reads. A cluster of reads is a group of reads that has some sequence overlap. Usually, reads with a length above a certain threshold are considered for further analysis to detect RBP binding sites. RBP binding site detection is a challenging process. Several computational methods such as WavClusteR, PARalyzer, Piranha, PIPE-CLIP, dCLIP, and GraphProt have been developed and utilized to detect RBP binding sites while minimizing the detection of false positives and background. Computational methods such Piranha and RIP-seeker have been developed specifically for peak calling [17]. Peak calling simply refers to a RNA sequence that interacts with a RNA-binding protein. The identification of peak calling is a crucial step that follows the alignment of the short-sequence reads to a reference genome. Piranha is mainly a peak-calling algorithm that provides a way to detect differential binding across different conditions. PIPE-CLIP is a Galaxy-based comprehensive online tool for CLIP-seq data analyses. It provides data processing but also statistical analyses [122]. PARalyzer is another popular peak calling algorithm for PAR-CLIP datasets only. Finally, motif recognition algorithms are used to identify motifs recognized by RBP. Algorithms such as MEMEris, PhyloGibbs, RNAcontext, and RNAmotifs identify motifs at the RNA level, while MEME, cERMIT, GLAM2, and MatrixREDUCE were developed to identify motif at the DNA level. Lastly, the identified RBP motif is analyzed using mCarts or GraphProt to predict candidate RBP binding sites within the same organism. A summary of computational methods employed for high-throughput RNA-protein interaction data has been provided by Bahrami-Samani *et al.* [17]. In addition, a recent review by Janga has summarized different computational and experimental methods employed to identify novel RBP and their targets [123]. Because of increasing use of genome-wide analyses and evolving computational tools, a staggering amount of data is being generated. Of course, such data sets are prone to detecting false positives due to: (1) replacement of non-covalent bonds by UV-induced covalent bonds that cannot be eliminated by stringent washing; (2) ultrasensitive nature of high-throughput sequencing; and lastly, (3) development of tools that fits one scientist’s needs and hence is not a universal analysis tool. Our next challenge will be to develop and refine bio-informatics tools that can be used easily by all researchers. However, perhaps the most crucial issue to be considered is the actual biological validation of the data. This important task is often laborious and time-consuming but rewarding.

## 7. Conclusions and Future Directions

The search for better and more selective drugs for the treatment of cancer, infections and many other pathologies is an ongoing and challenging effort. New discoveries in the RNA field open up opportunities for RNA-binding proteins to serve as therapeutic agents. When we have a better understanding of the targets of RNA-binding proteins, it will be possible to modulate gene expression at several post-transcriptional levels. Tailored RNA-binding proteins can be used to alter expression of single genes or even complete gene families whose transcripts share common “*cis* acting elements”. When efficient gene delivery systems become a reality RNA-binding proteins may become the “drugs” of choice for customized therapies.
